# The Inebriates' Acts, 1879—1899

**Published:** 1900-09

**Authors:** William Cotton

**Affiliations:** Hon. Med. Officer Royal Victoria Home for Inebriates, Bristol


### THE INEBRIATES' ACTS, 1879—1899.

BY

WILLIAM COTTON, M.A., M.D. Edin., D.P.H.,

*Hon. Med. Officer Royal Victoria Home for Inebriates, Bristol.*

---

THERE is no statutory enactment touching alcoholic or other inebriates (*A*), who by private agreement become resident patients in various Homes, or in the houses of medical men (1). There is no official cognisance or inspection of such, nor any form of legal restraint where moral influences fail.

The following are the Acts of Parliament relating to the "inebriate" or "habitual drunkard":

No. I.—An Act to facilitate the control and care of Habitual Drunkards (1879).

II.—An Act to amend the Habitual Drunkards' Act, 1879 (1888).

III.—An Act to consolidate the Acts relating to the Prevention of Cruelty to, and Protection of, Children (1894).

IV.—An Act to provide for the treatment of Habitual Inebriates (1898).

V.—An Act to Amend the Inebriates' Act, 1898 (1899).

An habitual drunkard means "a person who, not being amenable to any jurisdiction in lunacy, is notwithstanding, by means of habitual intemperate drinking of intoxicating liquor, at times dangerous to himself or to herself, or incapable of managing himself or herself, and his or her affairs." In a footnote to one of the forms now in use it is stated: "The Secretary of State (Home Secretary) is advised that 'intoxicating liquor' may include liquors other than alcohol, if their habitual intemperate use brings the consumer into the condition of an 'Habitual Drunkard.'"

Of the above Acts, Nos. I., II., and the amending parts of No. IV. refer to non-criminal inebriates voluntarily applying for admission (*B*) to a duly licensed "Retreat" (2). Observing certain formalities, they sign an application for admission to the licensee before any one Justice of the Peace, who attests their signature. By No. III., an habitual drunkard convicted of cruelty to a child may, in place of imprisonment, be sent with his consent (*C*) to a "Licensed Retreat," as if he were voluntarily applying in the ordinary way—this provision appears to be a dead letter. The rest of No. IV. and No. V. refer to criminal inebriates (*D* and *E*), who may, on conviction for certain offences, be compulsorily sent to and detained in "Inebriate Reformatories" established or certified by the Secretary of State. The Acts apply, *mutatis mutandis*, to Scotland and Ireland.

The Act of 1898 (No. IV.) introduces certain novel points: (1) As regards "Licensed Retreats," the license may be granted for a period up to two years in place of thirteen months. A patient may be detained up to two years in place of up to twelve months, and extension of his term and his readmission are simplified. By section 14, "The council of any county or borough may contribute such sums, and on such conditions as they may think fit, towards the establishment and maintenance of a retreat under the Inebriates' Acts, 1879 and 1888, as amended by this Act, and two or more councils may combine for any such purpose." The great remaining blots on the Act are, the necessity of the applicant for admission appearing before a justice at all, and the absence of power for a licensee to recover an escaped patient without a warrant and recourse to the police.

(2) As regards "Inebriate Reformatories" (the creation of the Act), two grades of criminal drunkards and two sets of reformatories are contemplated. By section 1, where a person is convicted of an offence punishable with imprisonment or penal servitude (*i.e.* any serious crime), if the court is satisfied from the evidence that the offence was committed under the influence of drink, or that drunkenness was a contributing cause of the offence, and the offender admits that he is, or is found by the jury to be, an habitual drunkard, the court "may," in addition to or in substitution for any other sentence, order that he be detained for a term not exceeding three years in any State Inebriate Reformatory (3) or in any Certified Inebriate Reformatory (4). By section 2, any person who commits any of the offences scheduled (*i.e.* different forms of disorder with drunkenness), and who within the twelve months preceding the date of the commission of the offence has been convicted at least three times of any offence so mentioned, and who is an habitual drunkard, "shall be liable" on conviction to be detained for a term not exceeding three years in any Certified Inebriate Reformatory.

The intention to establish a State Inebriate Reformatory (an Inebriate Broadmoor?) is for the present abandoned.

In reference to local institutions, the Royal Victoria Home, Brentry, is a "Licensed Retreat" for 50 females, and a "Certified Inebriate Reformatory" for 75 females and 30 males. The parent charity, the Royal Victoria Home, Horfield, was formerly a "Licensed Retreat"; it is now a "Certified Inebriate Reformatory" for 25 females, used as

### THE LEGISLATIVE CLASSIFICATION OF THE HABITUAL DRUNKARD.

| Institution. | Established and Maintained by | Number and Accommodation (May, 1900). | Nature of Case. | Admission. | Discharge. | Special Acts of Parliament, or parts thereof (Short Titles). |
| --- | --- | --- | --- | --- | --- | --- |
| (1) **Private Retreat, Home or House,** "not under the Act." | Any person or persons. | Uncertain. | (A) Voluntary private inebriate patient or boarder. | Private arrangement or contract. | Legally at the will of the patient. | None. |
| (2) **Licensed Retreat** "under the Act." | Any person or two or more persons jointly, licensed by a Borough or County Council. Any Council may contribute. | Number—17. Accommodation for 305 (131 males, 174 females). | (B) Habitual drunkard voluntarily applying. | Formal application to licensee by patient, with two statutory declarations that he is a habitual drunkard, applicant's signature being attested by any one Justice of the Peace. Maximum detention two years. | Expiry of period of detention: order of a Justice of the Peace or Judge, &c. | I. Habitual Drunkards' Act, 1879. II. Inebriates' Act, 1888. IV. Inebriates' Act, 1899, sections 13 to 21 inclusive, and part section 27, and sections 28 to 30 inclusive. |
| | | | (C) Habitual drunkard guilty of cruelty to a child—with his consent. | On conviction, Court may, in place of a sentence of imprisonment, make an order for his detention for a period not exceeding 12 months. | As next above. | III. Prevention of Cruelty to Children Act, 1894, section 11. |
| (4) **Certified Inebriate Reformatory,** *i.e.* Reformatory certified by the Home Secretary. | Any Borough or County Council or any persons desirous. Any Council may contribute. (To expenses of detention Treasury may contribute.) | Number—4. Accommodation for 208 (30 males, 178 females). | (D) Habitual drunkard guilty of an offence punishable with imprisonment or penal servitude. | After conviction the Court may, in addition to or in substitution for any other sentence, order that he be detained for not more than three years. To (3) or (4). | Expiry of period of detention and regulations of Home Secretary. | IV. Inebriates' Act, 1898, except sections 13 to 20 inclusive, part section 27, and section 28. |
| | | | (E) Habitual drunkard four times convicted of drunkenness as scheduled within the year. | After conviction shall be liable to be detained for not more than three years. | As next above. | V. Inebriates' Act, 1899. |

reception house for the other, no case being retained for more than twenty-eight days. Private patients ("not under the Act") of limited means have also been admitted at both Brentry and Horfield. The two Homes were the first to be certified as "Inebriate Reformatories" under the Act of 1898 (No. IV.), on March 30th, 1899.

For those inebriates who are unwilling to put themselves under any form of care and treatment, and who at the same time keep outside the pale of the law, except so far as they may be qualifying as "repeaters," there exists no form of legal compulsion, and, consequently, no institution is providable for them at present.

The appended table exhibits pretty fully the scope and limitations of the existing legislation. For the sake of simplicity, reference to a State Inebriate Reformatory (3) is omitted. This would be established and maintained by the Home Secretary out of moneys voted by Parliament, would admit Class D only, and to it the Prisons Acts (1865—1898) would apply.

